# Supplementary material for: The RECQL helicase prevents replication fork collapse during replication stress
Source: Life Sci Alliance. 2020 Aug 20;3(10):e202000668. doi: 10.26508/lsa.202000668 (PMC7441523; doi:10.26508/lsa.202000668)
Supplement: Supplementary file 1 [file LSA-2020-00668_TableS1.docx]

Supplemental Table 1: Overview of shRNA vectors.

| Cell line | shRNA vector | shRNA sequence |
| --- | --- | --- |
| TBP-RecqlKD MEFs | TRCN0000115248 | GCTTGTCTTCTCAGCAACGAA |
| TBP-RecqlKD#2 MEFs | TRCN0000115249 | GTGTGTTAATAGCACAGCATT |
| TBP-RecqlKD#3 MEFs | TRCN0000115250 | GATGGTGTCATACTGCCAGAA |
| RPE-empty-RecqlKD | TRCN0000289591 | GCACATGCTATTACTATGCAA |
| RPE-c-MYC-RecqlKD | TRCN0000289591 | GCACATGCTATTACTATGCAA |
