# Supplementary material for: The RECQL helicase prevents replication fork collapse during replication stress
Source: Life Sci Alliance. 2020 Aug 20;3(10):e202000668. doi: 10.26508/lsa.202000668 (PMC7441523; doi:10.26508/lsa.202000668)
Supplement: Supplementary file 2 [file LSA-2020-00668_TableS2.docx]

Supplemental Table 2: Overview of sequencing primers.

| Primer | Sequence |
| --- | --- |
| PCR1F1 | ACACTCTTTCCCTACACGACGCTCTTCCGATCTCGTGATGGCTTTATATATCTTGTGGAAAGGACG |
| PCR1F2 | ACACTCTTTCCCTACACGACGCTCTTCCGATCTACATCGGGCTTTATATATCTTGTGGAAAGGACG |
| PCR1F3 | ACACTCTTTCCCTACACGACGCTCTTCCGATCTGCCTAAGGCTTTATATATCTTGTGGAAAGGACG |
| PCR1F4 | ACACTCTTTCCCTACACGACGCTCTTCCGATCTTGGTCAGGCTTTATATATCTTGTGGAAAGGACG |
| PCR1F5 | ACACTCTTTCCCTACACGACGCTCTTCCGATCTCACTGTGGCTTTATATATCTTGTGGAAAGGACG |
| PCR1F6 | ACACTCTTTCCCTACACGACGCTCTTCCGATCTATTGGCGGCTTTATATATCTTGTGGAAAGGACG |
| PCR1F7 | ACACTCTTTCCCTACACGACGCTCTTCCGATCTGATCTGGGCTTTATATATCTTGTGGAAAGGACG |
| PCR1F8 | ACACTCTTTCCCTACACGACGCTCTTCCTCAAGTATCTGGGCTTTATATATCTTGTGGAAAGGACG |
| PCR1F9 | ACACTCTTTCCCTACACGACGCTCTTCCCTGATCATCTGGGCTTTATATATCTTGTGGAAAGGACG |
| PCR1R | GTGACTGGAGTTCAGACGTGTGCTCTTCCGATCTACTGACGGGCACCGGAGCCAATTCC |
| PCR2F | AATGATACGGCGACCACCGAGATCTACACTCTTTCCCTACACGACGCTCTTCCGATCT |
| PCR2R | AGCAGAAGACGGCATACGAGATATCACG GTGACTGGAGTTCAGACGTGTGCTCTTCCGATCT |
